# Melatonin Mitigates Mitochondrial Meltdown: Interactions with SIRT3

**DOI:** 10.3390/ijms19082439

**Published:** 2018-08-18

**Authors:** Russel J. Reiter, Dun Xian Tan, Sergio Rosales-Corral, Annia Galano, Mei-Jie Jou, Dario Acuna-Castroviejo

**Affiliations:** 1 Department of Cell Systems and Anatomy, UT Health San Antonio, San Antonio, TX 78229, USA; tan@uthscsa.edu; 2 Centro de Investigacion Biomedica de Occidente, Instituto Mexicano del Seguro Social, Guardalajara, 4436 Jalisco, Mexico; espiral17@gmail.com; 3 Departamento de Quimica, Universidad Antonoma Metropolitana-Unidad Iztapalapa, San Rafael Atlixco 186, Col. Vicentina, Iztapalapa, C.P. 09340 Mexico D.F., Mexico; agalano@prodigy.net.mx; 4 Department of Physiology and Pharmacology, Chang Gung University, 259 Wen-Hwa 1st Road, Kwei-Shan, Tao-Yuan 333, Taiwan; mjjou@mail.cgu.edu.tw; 5 Departamento de Fisiologia, Instituto de Biotecnologia, Universidad de Granada, Avenida de Conocimiento S/U, 18016 Granada, Spain; dacuna@ugr.es

**Keywords:** reactive oxygen species, oxidative stress, molecular pathways, sirtuins, antioxidant enzymes, oxidative phosphorylation

## Abstract

Melatonin exhibits extraordinary diversity in terms of its functions and distribution. When discovered, it was thought to be uniquely of pineal gland origin. Subsequently, melatonin synthesis was identified in a variety of organs and recently it was shown to be produced in the mitochondria. Since mitochondria exist in every cell, with a few exceptions, it means that every vertebrate, invertebrate, and plant cell produces melatonin. The mitochondrial synthesis of melatonin is not photoperiod-dependent, but it may be inducible under conditions of stress. Mitochondria-produced melatonin is not released into the systemic circulation, but rather is used primarily in its cell of origin. Melatonin’s functions in the mitochondria are highly diverse, not unlike those of sirtuin 3 (SIRT3). SIRT3 is an NAD+-dependent deacetylase which regulates, among many functions, the redox state of the mitochondria. Recent data proves that melatonin and SIRT3 post-translationally collaborate in regulating free radical generation and removal from mitochondria. Since melatonin and SIRT3 have cohabitated in the mitochondria for many eons, we predict that these molecules interact in many other ways to control mitochondrial physiology. It is predicted that these mutual functions will be intensely investigated in the next decade and importantly, we assume that the findings will have significant applications for preventing/delaying some age-related diseases and aging itself.

## 1. Introduction

Genetic diversity often exceeds that which is predicted based on the number of genes that have been sequenced [1,2]. This suggests that epigenetic processes may account for the greater molecular heterogeneity than that which is apparent in organisms [3,4]. Within recent decades, unraveling the mechanisms whereby the large variety of epigenetic modifications (post-translational modifications) are achieved has been a major area of research [5,6,7].

Sirtuins are a class of histone deacetylates that are homologues of a translational repressor, the first of which was originally discovered in yeast (*Saccharomyces cerevisiae*) and named mating-type regulator 1 (MAR1) [8,9]. Almost a decade later, Rine and colleagues [10] extended the findings of Klar et al. [8,9] when they identified four deacetylases in *S. cerevisiae*; they abandoned the name mating-type regulator and renamed the enzymes silent information regulators; since they had identified four such enzymes they were named SIR1-4.

In the last 25 years, the associated enzymes, now called sirtuins (SIRT), have been major agents of intensive investigative endeavor and they have been linked to a wide variety of physiological processes [11,12,13]. The actions include longevity-promoting effects [14,15], for their ability to inhibit cancer [16,17] and, more recently, because of their involvement in circadian rhythm regulation [18,19,20].

**Figure 1:**
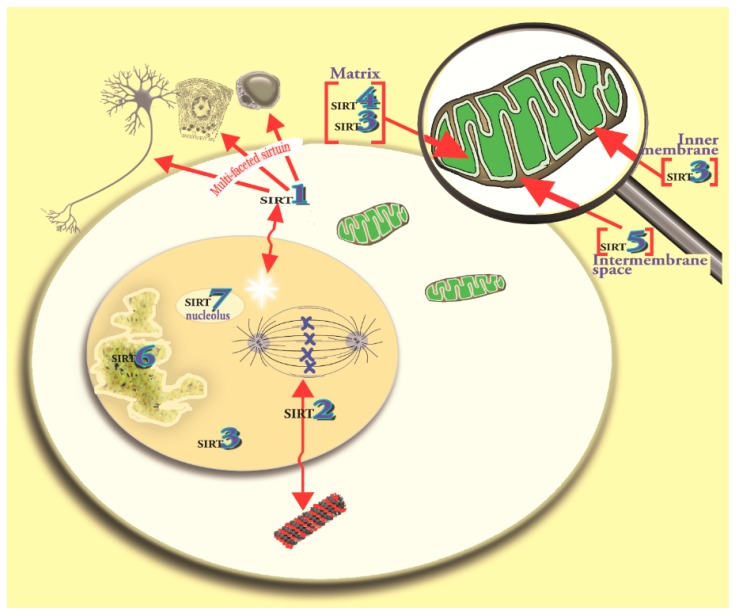
This figure identifies the primary location of sirtuins 1–7 in eukaryotes. Sirtuins are NAD+-dependent deacetylates which account for post-translational modifications of molecules within cells; sirtuins have a wide variety of functions including the promotion of longevity and the prevention in age-related diseases. For the purposes of the current report, specific interest is directed to mitochondrial SIRT3, the activity of which has been shown to be modulated by melatonin. SIRT3 is primarily located in the mitochondria where it co-localizes with melatonin. Both these molecules are involved in redox regulation and, as shown in this report, they are functionally intertwined. Single arrowheads identify structure; double headed arrows identify movement of molecules.

The sirtuin family in mammalian cells now includes seven members (SIRT1–7); they are collectively known as the cellular acylome [21]. Each sirtuin has a well-conserved NAD+-binding domain. The sirtuins have a variety of enzymatic activities and functions and they are differentially distributed at the subcellular level (Figure 1) [1,22]. One major function of sirtuins is to act as sensors of energy availability and, during each reaction, they consume molecules of NAD+ [5,23]. The best-known members of the sirtuin family that function as metabolic sensors include SIRT1, which is primarily located in the nucleus of cells and SIRT3 which is concentrated in the mitochondria [24,25]. Mitochondrial SIRT3 [12,26] is susceptible to regulation by exogenously-supplied melatonin; this is consistent with melatonin being a mitochondria-targeted antioxidant [27,28,29,30].

Of the seven sirtuins, three (SIRT3, SIRT4 and SIRT5) are located, although not exclusively, in the mitochondria, attesting to their potential involvement in their regulation of metabolism and especially of respiratory changes and oxidative phosphorylation [31,32,33]. Evidence has accumulated showing that melatonin, which exists in mitochondria along with SIRT3, influences the deacetylation of this sirtuin [28]. Since SIRT3 is known to modulate the redox state of the mitochondria, as does melatonin, we surmised these actions may be interrelated. Those relationships are discussed in this report.

## 2. Melatonin: Harnessing Free Radicals

The discovery of melatonin as a free radical scavenger 25 years ago [34] was preceded by reports that the indole protected against DNA damage as a result of being exposed to a chemical carcinogen [35] and was alleged to be a life-promoting molecule, as was SIRT3. Given that both these processes were believed to be related to oxidative stress [36,37], the initial studies designed to test the radical trapping ability of melatonin were undertaken. The hydroxyl radical (•OH), the most destructive of the oxygen-based toxic species, was generated by exposing a solution of hydrogen peroxide (H_2_O_2_) to ultraviolet light. A spin trapping agent, 5,5′-dimethylpyrroline N-oxide (DMPO), was added to the solution with or without the addition of melatonin; DMPO captures the •OH with the formation of an adduct, DMPO-•OH [38]. In the absence of melatonin in the solution, this adduct was abundant; however, when melatonin was added to the solution adduct formation was almost zero indicating melatonin had scavenged the •OH and reduced their availability to form a DMPO-•OH. When melatonin was compared to two other known •OH scavengers, glutathione or mannitol, melatonin proved superior as a radical quencher in this pure chemical system. The presence of the DMPO-•OH adduct was verified using high performance liquid chromatography with an electrochemical detector (HPLC-EC) and further confirmed with electron spin resonance spectroscopy (ESR) [39]. Given that these studies were performed using a cell-free system, it was obvious that the radical scavenging actions did not require membrane melatonin receptors (or any other receptor/binding site) which had been described [40,41] to exist in many cells.

The discovery that melatonin was a highly-efficient •OH scavenger was met with considerable skepticism by mainstream free radical biologists. They were resistant for several reasons. The first reason seemed to be was that they simply did not believe the data. Previously, there had been no hint that melatonin or related molecules had radical scavenging potential. Also, they argued that because of its very high reactivity, the •OH interacts (is scavenged by) with any molecule in its vicinity. In our study, however, we had compared melatonin with two confirmed •OH scavengers, i.e., glutathione and mannitol, both of which showed less reactivity to the free radical than did melatonin. Within a year after the initial publication, the radical scavenging capability of melatonin was confirmed in another chemical system using other methods [42].

Another reason that was used for rejecting the idea of the radical scavenging capability of melatonin was that there was little evidence that it functions in the detoxification and the reduction of oxidative stress in vivo or in cultured cells. This deficiency was soon rectified, however, since within two years melatonin was documented to reduce oxidative stress in vivo due to glutathione depletion [43], ionizing radiation exposure [44], paraquat toxicity [45], lipopolysaccharide [46,47], kainic acid [48], and ischemic/reperfusion injury [49]. The cellular damage in each of these situations is known to involve toxic free radicals and strongly support the idea that melatonin can protect a variety of molecules against oxidative stress in vivo.

The repeated independent verification of the capability of melatonin to function as a free radical scavenger [50,51,52,53,54,55,56,57,58,59,60,61,62,63,64,65,66,67,68] and to hold oxidative stress in check has solidified the idea that this intrinsically-produced indole is a legitimate in vivo agent that limits oxidative damage. An additional small percentage of the total number of published reports substantiating the ability of melatonin to limit oxidative stress under conditions that would have otherwise had disastrous consequences are summarized in Table 1 [69,70,71,72,73,74,75,76,77,78,79,80,81,82,83,84,85,86,87,88,89,90,91,92,93,94,95,96,97,98,99,100]. 

**Figure 2:**
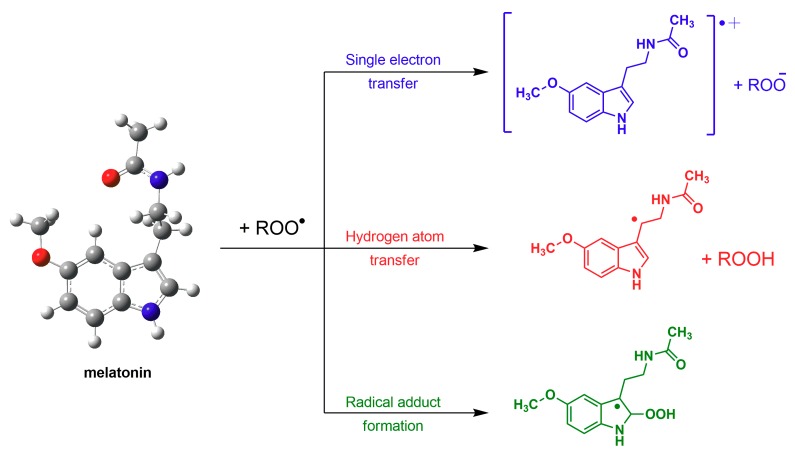
The means by which melatonin detoxifies a lipid peroxyl radical (LOO•) are shown in this figure and include electron transfer, hydrogen ion transfer and radical adduct formation. These are examples of actions of melatonin that are receptor independent. If the free radical was a •OH radical rather than a LOO• radical, because of the very high reactivity of the former, the number of reaction products would be much greater.

Some of the mechanisms by which melatonin detoxifies the peroxyl radical include (a) single electron transfer; (b) hydrogen atom transfer; or (c) possibly radical adduct formation [101,102,103] (Figure 2). When melatonin interacts with other radicals the adducts formed may be different than the one shown in the figure. Despite overwhelming evidence, there may be some who deny that melatonin is a radical scavenger or that it can curb oxidative stress [104,105] or they claim that it is only a weak scavenger [104,106].

Those who assert that melatonin is unlikely an essential physiological antioxidant in vivo argue that, because pharmacological quantities must be given to combat severe oxidative damage, e.g., during ischemia/reperfusion events, it is normally inconsequential as an inhibitor of oxidative stress. Oxidative damage would never occur if physiological concentrations of all antioxidants combined, i.e., vitamin C, glutathione, uric acid, melatonin, etc., were adequate to overcome the massive onslaught of free radicals that occur under such conditions. Thus, “pharmacological” amounts of free radicals are produced during these catastrophic events, which requires pharmacological concentrations of any antioxidant, not only melatonin, to effectively combat the molecular damage.

Other aspects of melatonin that contribute to its image as a highly-effective antioxidant include (a) metabolites that are formed when melatonin incapacitates a radical species also function in free radical detoxification [66,107,108,109,110,111,112,113] in what is called the antioxidant cascade; (b) melatonin chelates metal ions that are involved in the Haber-Weiss and Fenton reactions thereby preventing the formation of the devastatingly reactive •OH [114,115]; (c) melatonin stimulates antioxidant enzymes which removes potential damaging species from the intercellular environment [116,117,118,119,120,121,122,123] while; (d) inhibiting pro-oxidant enzymes (e.g., myeloperoxidase, lipoxygenase, etc.) [124]; (e) melatonin also synergizes with classic radical scavengers making them more effective in reducing oxidative damage [125,126]; (f) melatonin stimulates a cytosolic detoxifying enzyme quinone reductase [127]; and (g) melatonin increases the efficiency of the transfer of electrons between the mitochondrial respiratory complexes thereby reducing electron leakage and free radical formation [128,129]. Considering the multiple means by which melatonin annihilates reactive oxygen species, it would seem to be relentless in preventing molecular damage and maintaining a functional subcellular infrastructure.

## 3. Intrinsic Sources of Melatonin

Melatonin, although initially thought to be synthesized only in the pineal gland [130,131,132], was soon found to be produced in the vertebrate retinas as well [133,134]. Of these two sites, only the mammalian pineal gland is known to release significant amounts, presumably by simple diffusion out of the cells, of melatonin that would function systematically, with two routes of discharge being utilized, i.e., directly into the third ventricle of the central nervous system [124,135,136,137] and into the blood capillaries that are abundant in this tissue [138,139]. Although there may be some “incidental” leakage of small amounts of melatonin into the blood from other tissues (e.g., gut) there is no compelling evidence for a persistent or cyclic release of melatonin from these organs [140] as has been shown for the pineal gland [141,142]. If melatonin escapes the pinealocyte by simple diffusion into the blood, why melatonin produced in other cells does not do so as well is unexplained. Thus, following surgical removal of the pineal gland, circulating levels of melatonin persist at values that are barely measurable [141,142]. Exogenously administered melatonin, e.g., orally, subcutaneously, or intraperitoneally, does cause transient large increases in blood melatonin concentrations [143,144].

### 3.1. Melatonin in Bodily Fluids

**Figure 3:**
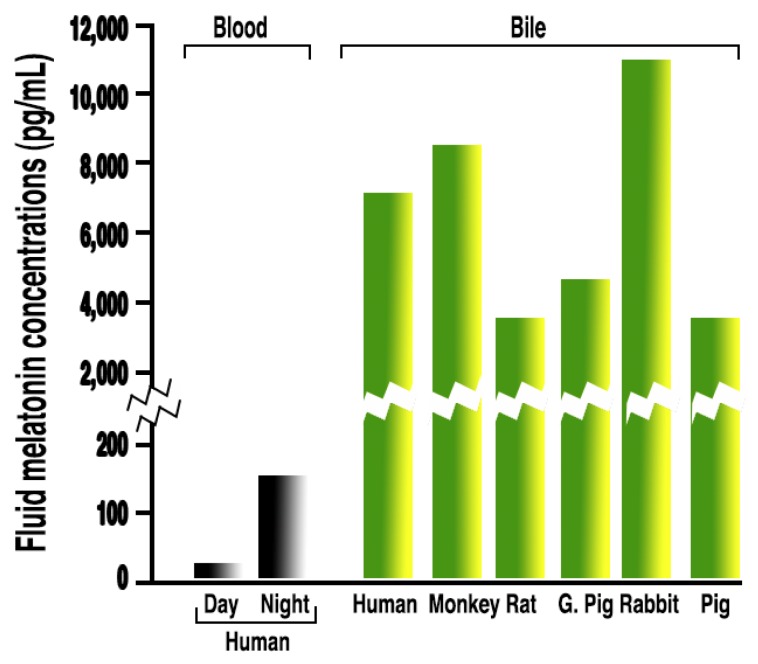
Radioimmunoassayable melatonin levels in the bile of six mammalian species. These values are clearly much higher than in the blood, both during the day or at night (two bars on far left). Levels of melatonin in the fluids other than the blood that exceed the concentration of this indole in the blood is not unusual. Melatonin values in several other bodily fluids and within subcellular organelles also exceed those in the blood.

Despite these general observations, based on the high melatonin concentrations in some bodily fluids, the possibility that other cells may produce and release melatonin into fluids other than the blood has been speculated, e.g., very high levels of melatonin in the bile (Figure 3) may be from hepatocytes [145] and concentrations of melatonin in ovarian follicular fluid that exceed those in the blood may be from granulosa cells [146,147] and perhaps from oocytes themselves [148].

In the pineal gland, melatonin is produced and released in a photoperiodic-dependent circadian manner [149,150] with the cerebrospinal fluid (CSF) and blood levels modulating, respectively, organismal-wide circadian rhythms via actions on the master biological clock, the suprachiasmatic nucleus (SCN) [151,152,153], and on peripheral oscillators that exist in presumably all cells [154,155,156,157]. How important the quantity of melatonin released from the pineal gland is relative to the total amount of oxidative stress an animal experiences, e.g., as a result of the persistent mitochondrial metabolism of oxygen and production of adenosine triphosphate (ATP), is not well defined [158,159,160]. Moreover, whether the elevated oxidative stress that occurs after surgical removal of the pineal gland is exclusively related to the loss of the nocturnal rise in melatonin or whether it is a consequence of circadian dysregulation remains undetermined. Any treatment that causes a perturbation of the melatonin rhythm is likewise associated with some change in circadian rhythmicity, and vice versa [161]. Thus, identifying which of those is the cause of a specific abnormality cannot always be deciphered. 

If the amount of melatonin that is released from the pineal gland is, in fact, relevant as a direct intracellular free radical scavenger in peripheral tissues, by necessity it would have to enter cells and gain access to mitochondria, the major site of reactive oxygen species (ROS) generation. Physiological levels of circulating melatonin have a short half-life in the blood (30–40 min) indicating it is rapidly taken up by peripheral tissues, either for metabolic breakdown (e.g., by hepatocytes) or by all tissues so it can carry out its intracellular functions (e.g., as a radical quencher).

Under extremely stressful conditions, e.g., forced swimming for rats, even high nocturnal levels of melatonin in the blood almost instantaneously disappear [162,163]; this very rapid disappearance seemingly exceeds the ability of the liver to catabolize such large amounts of melatonin in very short time. Rather, the swift drop in circulating blood melatonin concentrations under conditions of intensive stress more likely relates to its rapid uptake by cells throughout the body where it is needed to neutralize, directly or indirectly, the massive numbers of free radicals that are being produced, especially at the mitochondrial level [164,165].

### 3.2. Mitochondrial Melatonin 

**Figure 4:**
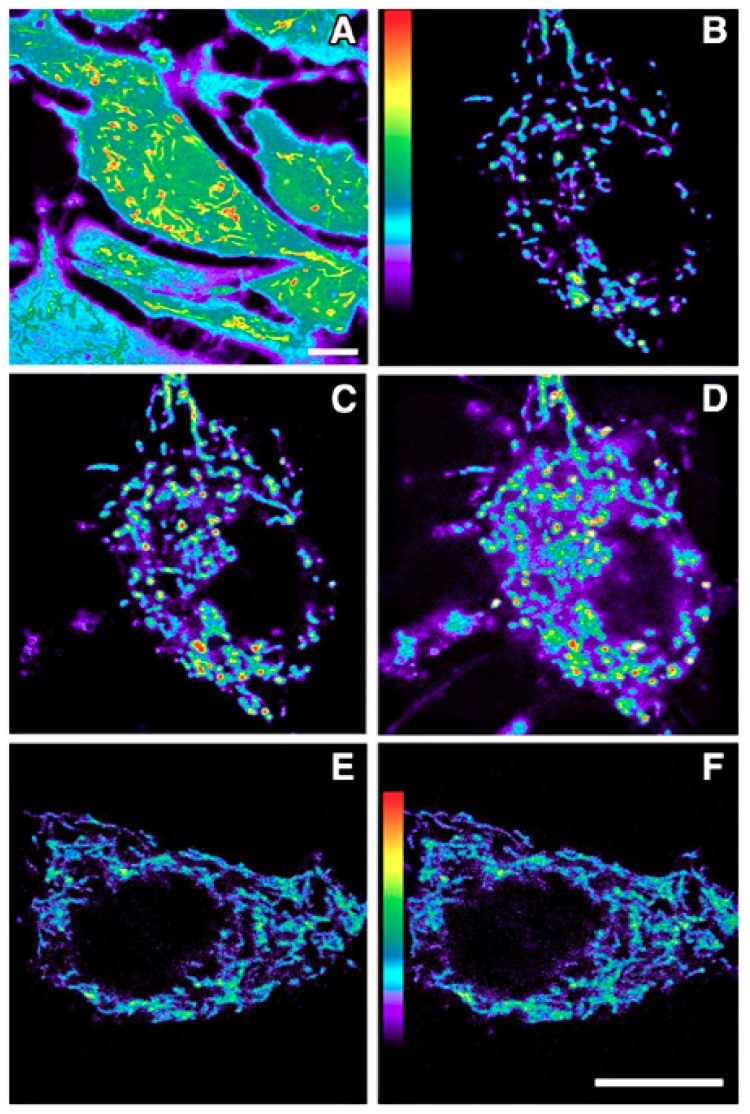
Fluorescent imaging of the intense generation of reactive oxygen species in a cultured astrocyte at 5 min (**C**) and 10 min (**D**) after its exposure to an oxidizing agent (H_2_O_2_). The cell shown in panels (**E**) (5 min) and (**F**) (10 min) was also exposed to the H_2_O_2_, but additionally, melatonin was present in the culture medium. Clearly, melatonin was taken into the cell and into mitochondria where it reduced reactive oxygen species generation or quickly scavenged them when they were produced. Dihydrorhodamine 123 was used to visualize free radical generation. (**A**) is an enhanced pseudocolor image which shows the higher levels of reactive oxygen species in mitochondria relative to other organelles. (**B**) is the astrocyte (also shown in the panels (**C**,**D**)) before its exposure to H_2_O_2_. Figure provided by Mei-Jie Jou. Scale bar = 1 µm.

That melatonin can enter cells and their mitochondria is undisputed. Using fluorescence imaging techniques, Jou and her colleagues [166,167,168] have repeatedly shown that high free radical generation by mitochondria is quickly quenched when the cells are incubated in a solution containing melatonin (Figure 4). These findings are consistent with melatonin from the incubation medium quickly penetrating the membranes of both the cell and of the mitochondria where it neutralizes, either by direct scavenging or via indirect means, the free radicals formed in the intermembrane space and in the matrix. Besides functioning as a radical scavenger in mitochondria, melatonin has other actions that probably necessitate its presence in these organelles. Thus, at this level, melatonin enhances mitochondrial antioxidant enzymes, promotes respiration and ATP production, negates the intrinsic apoptosis pathway, and mediates its effects on sirtuins [1,169,170]. These actions would generally require more time to appear since they would involve induction mechanisms.

**Figure 5:**
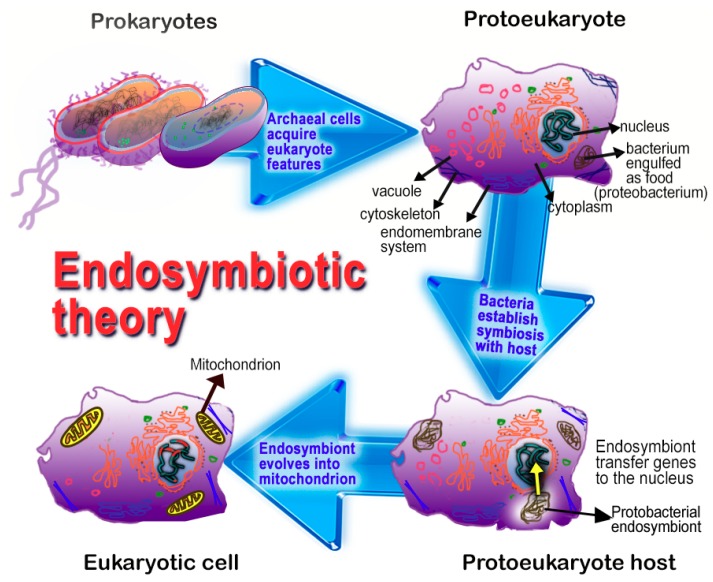
A summary of the major theory, the Endosymbiotic Theory, that explains the evolution of mitochondria as they exist in current-day eukaryotic cells. Accordingly, prokaryotic “cells” evolved features of eukaryotes over millions of years. An example of one major development, as it relates to the current review, is the phagocytosis of proteobacteria by prokaryotes; the bacteria were initially engulfed as nutrients by their digestion in intracellular granules. Eventually, the engulfed bacteria established a mutually beneficial association (a symbiotic relationship) with the host cell; the new endosymbiont sent much of its genetic material to the nucleus of the host while the symbiont gradually evolved into a mitochondrion. Since the proteobacteria already had the capability to produce energy (ATP) and melatonin, these functions were retained by the mitochondria and proved beneficial to the host cells. As a result, it is our contention that every mitochondria-containing cell produces melatonin in their mitochondria. The Endosymbiotic Theory for the origin of mitochondria was suggested more than 100 years ago and has been refined, and widely accepted, in the last three decades. Using current molecular technologies, it has been possible to document many metabolic similarities between bacteria and mitochondria. The Endosymbiotic Theory is used as an explanation as to why mitochondria produce melatonin, a function inherited from their precursors, i.e., bacteria.

To explain the ever-widening array of cells that synthesized melatonin, we recently proposed that multicellular organisms actually synthesize melatonin in their mitochondria [171]. This hypothesis is based on considerations related to the proposed origin of mitochondria in eukaryotes. According to the Endosymbiotic Theory, mitochondria in eukaryotes are derived from bacteria which were initially engulfed for their nutrient value; eventually, the phagocytosed bacteria developed a symbiotic relationship with their host cells and evolved into mitochondria (Figure 5) [172,173]. During evolution, the newly-developed mitochondria kept the important functions that their precursors possessed. Thus, they retained their ability to carry on oxidative phosphorylation and ATP production and their capacity to synthesize melatonin [28,171]. While bacteria have long been known to produce ATP as an energy source, only recently have bacteria been shown to contain melatonin, presumably a result of synthesis in these organisms [174,175,176]. Thus, any cell (animal or plant) that contains mitochondria may also have the capability to produce melatonin [28,171] since mitochondria derived from melatonin-producing bacteria [174,175]. The production of melatonin by mitochondria is now supported by reports that have been published after mitochondria being a source of melatonin was suggested (see below).

**Figure 6:**
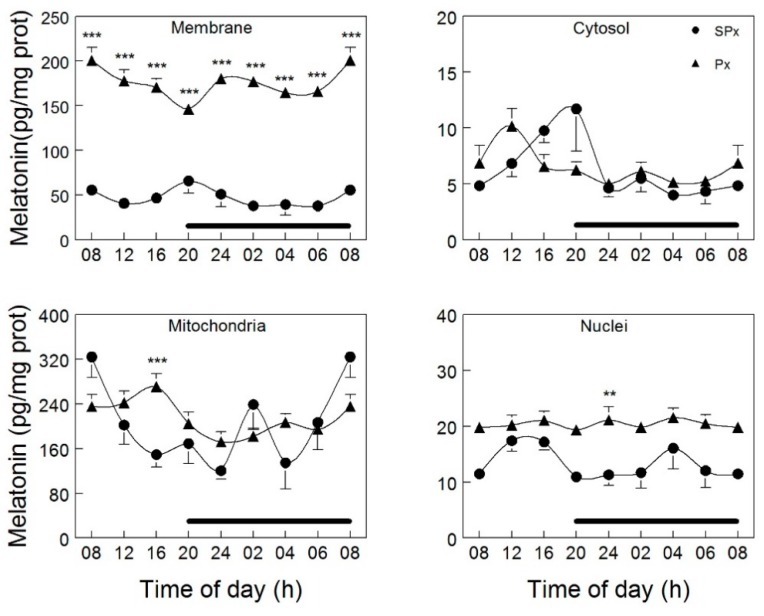
Concentrations and fluctuations of melatonin in organelles obtained from cells of the cerebral cortex of rats that were either intact (SPx) or pinealectomized (Px). Only in cellular membranes did Px markedly change melatonin levels; in this organelle, melatonin values rose substantially as a consequence of pineal removal (*p* < 0.001). In the mitochondria and nucleus, at one time point each, melatonin levels were elevated (** *p* < 0.05 and *** *p* < 0.001 respectively). Tissues were collected at 4 h intervals over a 24-h light:dark cycle. The period of darkness is identified by the horizontal black bar. What is most apparent is that levels of melatonin in brain mitochondria greatly exceed its values in other subcellular locations. Figure provided by Dario Acuna-Castroviejo.

In 2012, Venegas and coworkers [177] collected the brain and liver from rats at 4-h intervals over a 24-h light:dark cycle. For the purposes of determining whether melatonin was differentially distributed in neurons and hepatocytes and, if so, whether the concentrations varied between the light and dark periods, they isolated cellular membranes, cytosol, mitochondria, and nuclei. The results unequivocally show that, at least for brain cells and hepatocytes, melatonin levels are not in equilibrium at the subcellular level. Of the four fractions in which melatonin was measured, brain mitochondria had much higher melatonin concentrations than the other sites; thus, the levels of melatonin were about 25 times greater in mitochondria than in cytosol (Figure 6). When hepatocyte-derived mitochondria were examined, these values were not at the same high level as those seen in brain mitochondria.

The intracellular melatonin levels were clearly not derived from the blood since when these measurements were made in cells that were obtained from pinealectomized animals, a procedure which reduces circulating melatonin levels to essentially zero, the concentrations of melatonin actually increased in the membranes (10-fold) and cytosol (2-fold), but was essentially without influence on these levels in either the brain nuclei or mitochondria [177]. In both the intact and pinealectomized animals, organellar levels of melatonin did not vary over a 24-h period. The exceptionally high levels of melatonin in brain mitochondria speaks to the likelihood that this organelle is uncommonly efficient in sequestering/retaining melatonin or it produces it. The remarkably high concentrations of melatonin in brain mitochondria is fortuitous considering that this organelle produces many free radicals during respiration. This is supported by the fact that the central nervous system has a very high metabolic rate and uses a much larger portion of the inhaled oxygen than would be predicted based on its size.

The observations of Venegas et al. [177] do not preclude the possibility that mitochondria may both avidly take up melatonin from extracellular locations, e.g., blood, as well as synthesize it. As shown above, the reports of Jou et al. [167,168] document that when cultured cells are incubated with melatonin, it may rapidly gain access to mitochondria where it visually lowers free radical products. Similarly, also as mentioned above, melatonin given to animals via any route reduces reactive oxygen species damage to cells implying that the indole gets to the source of the bulk of the free radicals, i.e., the mitochondria.

It has generally been assumed that, as noted above, due to the high lipophilicity of melatonin, it readily enters cells via simple diffusion. While this remains a possible route for its entrance into cells, recent studies have examined active transport means by which it passes through membranes. The first serious attempt to determine how high intracellular levels of melatonin are achieved, Hevia et al. [178] examined the uptake of melatonin in a variety of normal and cancer cells. For this purpose, cells were incubated in a solution containing pharmacological concentrations (1 mM) of melatonin. Independent of the cell type, incubation for 24 h always increased intracellular levels of the indole. The findings were not consistent with the passive uptake of the indole; rather the data suggested a protein-mediated uptake process which was impacted by the ambient glucose concentration. The protein involved was subsequently identified as the GLUT1 transporter. Accelerated melatonin uptake is further exaggerated in a high glucose environment in erythrocytes [179]. 

Huo and colleagues [180] recently defined another means by which melatonin may enter both cells and mitochondria; in this case, however, only human cancer cells were examined. The results are consistent with the involvement of the PEPT1/2 oligopeptide transporters in the transfer of melatonin through both the cell and mitochondrial membranes. Docking analysis studies as well as kinetic measurements support a role for the oligopeptide transporters aiding the uptake and retention of melatonin within cells and especially within mitochondria. For a detailed discussion of the active transport mechanisms for melatonin, the reader is directed to a recent report by Mayo et al. [1].

In view of the seemingly special association of melatonin with mitochondria [27,171] along with the protective advantages against free radicals that this would provide, interest in the potential local synthesis of melatonin by mitochondria has become a subject of active investigation. Considering that melatonin-producing bacteria [174,175] are widely accepted to be the progenitors of eukaryotic mitochondria (see Endosymbiotic Theory mentioned above and in Figure 5), it seems likely that this important function would have been retained by eukaryotic cells as we have proposed [171].

### 3.3. Mitochondrial Synthesis of Melatonin 

In addition to the rapid uptake of melatonin by mitochondria, as noted above, we have recently drawn attention to the unique relationship of melatonin with mitochondria [27] and we proposed that this organelle likely produces its own melatonin [171]. Despite this, some toxic radicals escape being neutralized and damage adjacent macromolecules. The idea that mitochondria synthesize melatonin is based primarily on the predicted origin of mitochondria from bacteria, there is one very early study that also alluded to this possibility. In 1975, Kerenyi et al. [181] while examining the subcellular distribution of the rate-limiting enzyme in melatonin synthesis, arylalkylamine *N*-acetyltransferase (AANAT), claimed that in pinealocytes the reaction product for this enzyme is exclusively limited to the mitochondria. While the images of the mitochondria in this ultrastructural study are rather fragmented, nevertheless, the author’s interpretation of the findings cannot be disputed; the reaction product is in mitochondria. The results of the Kerenyi et al. [181] study were, however, rarely acknowledged in the subsequent years since individuals working on the synthesis of melatonin were likely convinced that this process was relegated to the cytosol and, also, the cytochemical method used to identify AANAT activity was less than optimal. The findings reported in the Kerenyi et al. [181] report were, however, used to support the argument of Tan et al. [171] that mitochondria are the primary site of the conversion of serotonin to melatonin. 

He et al. [148] recently examined mouse oocytes as a likely location of melatonin synthesis. In an electron micrographic study, immunocytochemical AANAT was restricted to the mitochondria. More importantly, when they incubated isolated oocyte mitochondria with serotonin, a necessary precursor of melatonin production, melatonin levels were markedly increased in the mitochondria as well as in the medium in which they were incubated; in the absence of serotonin no melatonin was formed. These findings have implications beyond the identification of melatonin in oocyte mitochondria. In all offspring of fertilized ova, the mitochondria are exclusively of maternal origin. Thus, it seems likely that since oocyte mitochondria produce melatonin, all cells in the offspring would also do so. 

A more complete study to establish where in cells melatonin formation occurs was published by Suofu and colleagues [182]. They used non-synaptosomal brain mitochondria for this purpose. The two terminal enzymes in melatonin production, AANAT and HIOMT, along with the AANAT chaperone, 14-3-3ζ, were in mitochondria. Like Venegas et al. [177], when melatonin synthesis was measured over a 24-h light:dark cycle, no rhythm in any of the parameters was apparent. Digesting the outer mitochondrial membrane with a combination of proteins K and digitonin did not lead to a loss of enzyme activities prompting the authors to conclude that melatonin synthesis likely occurs in the matrix. Finally, when brain mitochondria were incubated with deuterated (D4) serotonin, they yielded D4-*N*-acetylserotonin and D4 melatonin. The findings of Suofu et al. [182] are the most complete data indicating that mitochondria are a location of melatonin synthesis.

While the findings of Venegas et al. [177] and Suofu and coworkers [182] are noteworthy, in both reports the organ of greatest concern was the brain. In this organ, melatonin concentrations in mitochondria were very high [177] and the proteins of the melatonin-forming enzymes, AANAT and HIOMT/ASMT, were also present [182]. Thus, the presumed production of melatonin in neural tissue, of which the pineal is a part, may be unique to this organ and awaits identification in other tissues. However, based on the findings of He et al. [148] on the production of melatonin by oocyte mitochondria along with the organism-wide distribution of mitochondria from the oocyte, we feel that the mitochondria of all cells manufacture melatonin, possibly in tissue specific amounts as needed, i.e., melatonin may be inducible.

Melatonin solicits a large variety of auxiliary means to reduce mitochondrial damage potentially inflicted on hapless macromolecules as a consequence of the persistent low-level free radical generation or, in the case of toxin exposure, hypoxia, etc., to the torrents of ROS that are produced. Some of the allied processes utilized by melatonin to reduce these catastrophic effects, e.g., indiscriminate molecular destruction which ultimately leads to cellular apoptosis, include improving the efficacy of the mitochondria respiratory complexes [129,183,184], enriching ATP production [185,186], reducing free radical generation by limiting electron leakage, commonly referred to as radical avoidance [128], upregulating antioxidative enzymes [187,188], stimulating glutathione synthesis [189,190], direct radical detoxification [103,191] and probably yet to be defined means. The outcome of these combined actions is the preservation of cellular and organ physiology, as well as the prevention of organismal death, by mitigating cellular loss due to apoptosis [167,192,193,194,195].

## 4. The Melatonin/SIRT3 Interactions

Many post-translational modifications are mediated by sirtuins [6,7]. SIRT3 is a member of a family of seven sirtuins (1–7) that are collectively referred to as the cellular acylome [21]. Sirtuins mediate post-translational modifications of lysine residues; these actions are reversible and involve proteins in a wide variety of molecular pathways. As such, the sirtuins have a major impact on the physiology of cells [21,196,197,198,199,200]. Like melatonin, SIRT3 has actions related to reducing oxidative stress. For example, both melatonin and SIRT3 influence the generation of partially reduced oxygen species at the level of the mitochondrial respiratory complexes and both detoxify these reactants by promoting antioxidant enzyme activities [122,123]. Forkhead box 03 (FOXO3a) is a direct target of SIRT3 which is also involved in processes related to defense against oxidative damage [201,202], not unlike the actions of melatonin. Considering the common functional features of melatonin, FOXO3a and SIRT3 in mitochondria, their physiological association would seem to be a logical assumption.

### 4.1. Reproductive Organ Protection

Considering that human females are planning pregnancies later in their reproductive life span, there is increasing interest in identifying molecules that maintain ovarian, oocyte and uterine physiology in a more youthful state to ensure a successful pregnancy and healthy offspring. The human female reproductive axis typically declines quickly in women after the age of 35. Since, as in other organs, free radical damage contributes to diminished reproductive processes including the onset of menopause [203,204], the antioxidant melatonin has been tested for its potential in deferring the slowed follicular maturation and reduced oocyte quality observed in the aging female. The results of these studies verify that melatonin has significant benefits in maintaining the function of the ovary and adnexa which normally deteriorates with age or after exposure to toxins, etc. [205,206,207,208,209].

**Figure 7:**
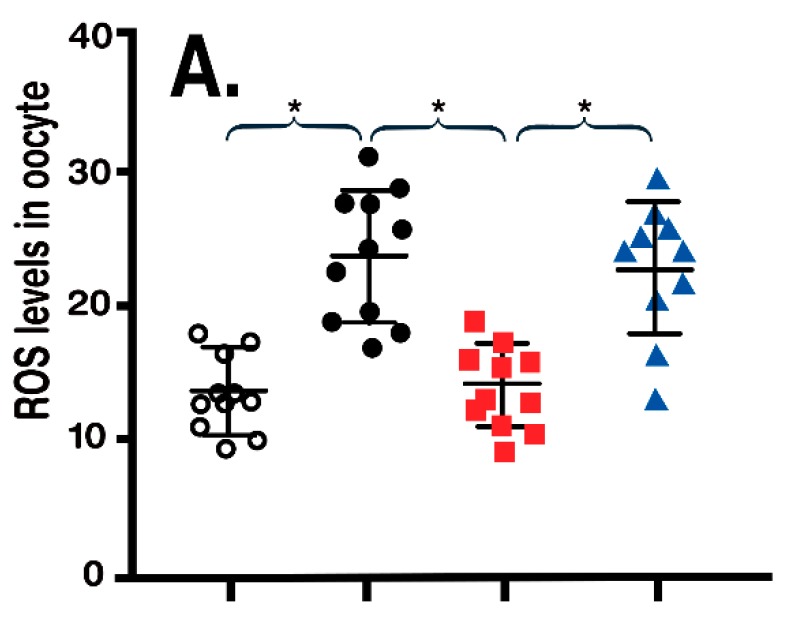

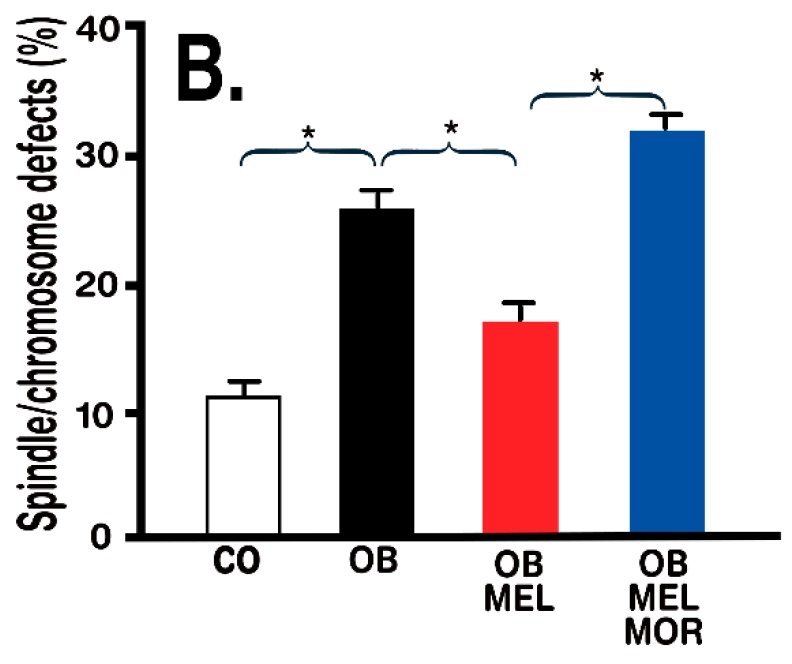
Reactive oxygen species (ROS) levels in oocytes of normal control (NO) and obese (OB) mice (**A**) and mean spindle/chromosomal defects (**B**) of the oocytes (values are means ± SEM). Obesity was associated with an increase in ROS and elevated molecular damage in oocytes. Melatonin (MEL) treatment reduced both parameters. When SIRT3 was silenced (with siRNA) the actions of melatonin were negated. This suggests that the antioxidant action of melatonin in oocytes depends on stimulation (deacetylation) of SIRT3 which enhances the activity of the antioxidant enzyme, superoxide dismutase. Figure drawn from the data of Han et al. [210]. * *p* > 0.001.

Obesity in rodents, as in humans, due to feeding/consuming a high fat diet (HFD) causes the acetylation of SIRT3, which reduces its activity and causes the catastrophic cellular damage due to unrestrained ROS generation. This damage occurs in many tissues including the oocyte, which, because of its diminished quality, jeopardizes successful fertilization and pregnancy. Because of the worldwide increase in obesity, female fertility has shown a marked decline. Since mitochondrial SIRT3 is suppressed in obese rodents, which leads to reduced SOD2 and unrestrained ROS formation, Han et al. [210] fed melatonin to obese rats. As anticipated, melatonin greatly improved oocyte quality and fertilizability; this was accompanied by an activation (deacetylation) of SIRT3, which induced SOD2 ultimately reducing oxidative stress in the oocytes. To further confirm the mechanism by which melatonin improved oocyte quality in obese mice, we used morpholino to knockdown SIRT3, a procedure that eliminated the protective actions of melatonin at the oocyte level (Figure 7).

When melatonin was orally administered chronically to aging female mice and rats, clearly their reproductive function and performance were improved relative to those in age-matched controls not treated with melatonin. In mice, the improvement was obvious in the maintained follicle pool, litter size after mating and telomere length as well as oocyte number and quality [211]. Similarly, in female rats, melatonin delayed ovarian aging as estimated by many of the same parameters [212] measured in mice. Both Song et al. [211] and Tamura and coworkers [212] predicted that SIRT3 upregulation would be related to the melatonin maintenance of ovarian physiology in the aged rodents. In mice, melatonin treatment was accompanied by enhanced ovarian granulosa cell SIRT3 activity along with the translocation of FOXO3a to the nucleus where it bound to the promoters of SOD2 and catalase (CAT) [211]. Both SOD2 and CAT are mitochondrial antioxidant enzymes. The mitochondria of the granulosa cells also less easily generated ROS due to the H_2_O_2_ treatment when melatonin was present. In addition to the upregulation of sirtuins, melatonin may defer ovarian aging due to its direct radical scavenging activities, stimulation of telomerase, reducing autophagy, etc.

The data showing that melatonin reduces free radical generation/damage in mitochondria along with the findings of several groups which indicate that this organelle rapidly takes up and synthesizes melatonin is of great interest; however, the specific mechanisms by which the indole limits oxidative damage to this organelle remains to be determined. As already noted, melatonin has a plethora of means to curtail molecular damage resulting from exposure to free radicals. Which accounts for the reduced oxidative stress along with the subcellular mechanisms involved requires definition.

### 4.2. Cardiovascular Protection

**Figure 8:**
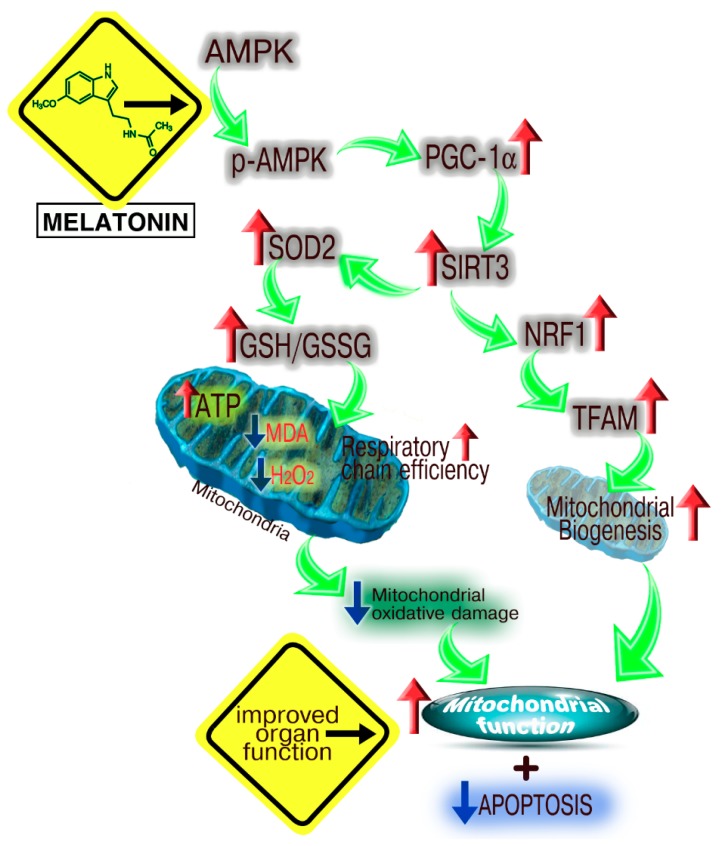
A summary of the proposed mechanisms by which melatonin preserves myocardial cell function after ischemia/reperfusion injury in type 1 diabetic rats. The involvement of AMPK→pAMPK was shown using compound C, a specific blocker of AMPK-signaling. The role of SIRT3 was documented with the use of SIRT3 siRNA. Melatonin protects against myocardial damage due to ischemia/reperfusion as indicated. Figure drawn from the findings of Yu et al. [216]. Red arrows indicate stimulation; blue arrows indicate inhibition; green arrows indicate flow of information.

There are numerous published reports showing that melatonin reduces ischemia/reperfusion (I/R) injury in multiple organs [213,214,215] (see also some data in Table 1). One I/R model involving the heart was used to examine the possible involvement of mitochondrial SIRT3 upregulation in melatonin’s protective actions in myocardial tissue subjected to anoxia/reoxygenation. Yu and colleagues [216] tested this in type 1 diabetic rats (induced by streptozotocin) after left anterior descending coronary artery ligation for 30 min. This study was specifically directed to examining the action of melatonin at the mitochondrial level. This comprehensive investigation validated that melatonin in I/R-damaged myocardium improved cardiac physiology by augmenting ATP production and improving respiratory chain function (Complexes II, III, and IV), stimulating SOD2 activity, limiting apoptosis of cardiomyocytes and reducing H_2_O_2_ generation and lipid peroxidation. Mechanistically, Yu et al. [216] found that melatonin’s beneficial effects stemmed from its stimulation of the adenosine monophosphate–activated kinase (AMPK)-PGC-1α-SIRT3 signaling and the activation of mitochondrial superoxide dismutase (SOD) and elevated NRF (nuclear factor (erythroid-derived 2)-like 2) and TFAM (mitochondrial transcription factor A) expression (Figure 8).

Two subsequent reports also confirmed the involvement of melatonin and SIRT3 in protecting the heart from ROS injury [217,218]. Based on the findings of Zhai et al. [217], the role played by SIRT3 was shown by the observation that a selective inhibitor of SIRT3 [3-(1H-1,2,3-triazol-4-yl) pyridine, 3-TYP) negated the effects of melatonin as a cardioprotective agent. Due to the depression of SIRT3, SOD2 is not upregulated and oxidative stress remains at a high level which severely compromises melatonin’s ability to protect the myocardium from free radical destruction.

Zhang et al. [218] used a different model to judge how effective melatonin is in protecting the myocardium from free radical damage. The model was the diabetic cardiomyopathic mouse. Heart function was monitored echocardiographically; these measurements showed that melatonin mitigated left ventricular cardiac remodeling and preserved cardiac function in diabetic animals. The authors suspected melatonin’s advantage resulted from its actions at the mitochondrial level. To prove this, Zhang et al. [218] examined the involvement of SIRT3; this confirmed that melatonin inhibited Mst phosphorylation which led to SIRT3 stimulation and elevated antioxidant protection. Melatonin’s efficacy disappeared in *Mst* knockout mice. They concluded that, as others have, that the upregulation of SIRT3 is essential to the ability of melatonin to protect mitochondria from oxidation stress.

Endothelial cell dysfunction in coronary arteries and elsewhere is a feature that is essential for the development of atherosclerotic lesions [219]. While the basic process involves inflammation of the endothelial lining, the associated oxidative stress including oxidation of low-density lipoprotein are important mediators of endothelial cell malfunctions eventually culminating in plaque formation and other subsequent negative effects [220]. Previously, melatonin was shown to maintain the optimal fluidity of cell membranes [221,222] and to protect them from damage normally inflicted by free radicals. 

Against this background of information, Lee et al. [223] examined the efficacy of melatonin to forestall endothelial cell damage in an ischemic limb, after lipopolysaccharide treatment and in an in vitro model using human umbilical vein endothelial cells (HUVECs). As anticipated, melatonin protected against endothelial cell destruction in the in vivo model and reduced oxidative stress levels, inflammatory reactions, cell death and mitochondrial dysfunction in HUVECs. Of special interest to the current report, when mitochondrial SIRT3 was silenced (with siRNA), melatonin’s in vitro effects were absent indicating that, as in other reports, melatonin’s antioxidant actions in mitochondria rely, to a large degree, on SIRT3 deacetylation. The authors point out that the reversal of the actions of melatonin by silencing SIRT3 are as others have observed in earlier reports. They do not provide, however, any additional information as to the signaling mechanisms by which melatonin modulates SIRT3/oxidative stress/mitochondrial malfunction.

### 4.3. Hepatic Protection

Fluorosis is a condition that occurs as a consequence of the long-term ingestion of fluoride. It is a progressive degenerative condition which is, in part, a result of exaggerated ROS production in hepatocyte mitochondria [224]. Melatonin has been successfully used to combat liver damage related to chronic ingestion of fluoride by experimental animals [225]. The mechanisms of protection afforded by melatonin involve limiting oxidative stress. Song et al. [226] showed that incubating human liver cells (L02) with sodium fluoride (NaF) increased intracellular superoxide production leading to the accumulation of oxidative stress and apoptotic cell death; these changes were attenuated when melatonin was added to the incubation medium. At the molecular level, melatonin stimulated SIRT3 deacetylation leading to the rapid dismutation of the superoxide; this was accompanied by SIRT3-triggered DNA binding of FOXO3a. The inhibitory pathway analysis showed that melatonin enhanced PGe-1α expression due to the activation of PI3K/AKT signaling. With the use of membrane melatonin receptor blocker, these workers proved the effects of melatonin were mediated by the MT1 receptor; this was confirmed when the L02 cells were transfected with MT1 siRNA. Presumably, the MT1 receptor involved was in the plasma membrane although it may have been in the outer mitochondrial membrane [182].

Chen and coworkers [227] examined the association of melatonin and SIRT3 on oxidative damage induced in cultured L02 hepatocytes and in mice after ligation of the bile duct. L02 is a spontaneously immortalized cell line. For the cell culture study, the active biliary toxin was glycochenodeoxycholic acid (GCDCA). Co-treatment of hepatocytes with melatonin and GCDCA highly significantly reduced mitochondrial ROS generation that was apparent in cells only treated with GCDCA. The protective actions of melatonin were associated with an upregulation of SIRT3 and the elevated deacetylation of SOD2. The actions of melatonin were blocked in hepatocytes transfected with AMP-activated, alpha-1 catalytic subunit (AMPK) siRNA. In the in vivo studies where the bile duct was ligated to produce cholestasis, the findings were similar. Melatonin’s beneficial actions in these studies were most likely related to its ability to modulate the AMPK-SIRT3-SOD2 pathway. The in vivo results are consistent with the ability of melatonin to reduce gross liver pathophysiology resulting from cholestasis due to obstruction of the bile duct [228,229].

## 5. Concluding Remarks

In recent years, investigations related to melatonin’s actions in cells have become progressively more focused to the mitochondria without, however, precluding functions in other subcellular compartments. Since melatonin is a powerful inhibitor of oxidative stress, its actions at the level of the mitochondria may not only be fortuitous, but necessary considering its highly protective actions. Mitochondria, more than any other organelle, spawns ROS/free radicals even under so-called “resting” conditions, but especially under taxing circumstances. It is essential that potentially-damaging ROS be quelled at their source to ensure the molecular damage they mete out can be averted.

More than a decade ago, it was immunocytochemically shown that melatonin quenched the fluorescent-signal associated with ROS generation in the mitochondria [167]. Roughly a decade later, melatonin was shown to be in exceptionally high concentrations in mitochondria, more so than in any other cellular compartment [177]. Among several interesting features of the Venegas et al. [177] report was that the mitochondria, in which the very high levels of melatonin were measured, were obtained from brain tissue, rather than being of pineal gland origin. After consideration of the preceding data, we speculated that melatonin is likely produced in the mitochondria of all cells, animals, and plants [171]. This hypothesis relies heavily on what is referred to as the Endosymbiotic Theory (Figure 5) for the origin of mitochondria in eukaryote cells. Since bacteria contain melatonin, presumably a result of its synthesis in these prokaryotes [174], and mitochondria derived from bacteria that were initially phagocytized as food by early eukaryotes, we surmised that the melatonin-synthesizing machinery of bacteria was retained by the evolved mitochondria. This idea stimulated investigations that have led to compelling evidence that mitochondria of every cell fabricates melatonin for its own use [145,182]; this explains the very wide distribution of melatonin in multicellular organisms [230,231]. That mitochondria produce melatonin has only been shown in a small number of cells, i.e., brain and oocyte. Whether this is a universal feature of mitochondria awaits investigation.

The original studies by Jou et al. [167,168] also suggested that, in addition to manufacturing melatonin, it is rapidly taken up by cells from the extracellular environment. This entry was thought to be related to simple diffusion of melatonin, which is highly lipophilic, through the plasma and mitochondrial membranes. While this possibility is still viable, melatonin has been shown to enter cells via the GLUT1 transporter [178] and by means of the PEPT1/2 oligopeptide transporters [180]. The latter proteins also exist in mitochondrial membranes where they presumably aid in the rapid uptake of melatonin against a gradient. It is anticipated that data related to the uptake and synthesis of melatonin by mitochondria will grow rapidly in the next five years. 

Like melatonin, sirtuins are both ubiquitously distributed and likely acquired by eukaryotes after the engulfment of α-proteobacteria [232]. As with melatonin, the sirtuins are therefore very ancient. The fact that both melatonin and sirtuins co-existed in cells for eons, it could be expected that they would have “learned” to collaborate. The data summarized above clearly shows that SIRT3 is required for melatonin to neutralize oxidative stress at the mitochondrial level, at least in the conditions under which it was investigated. These findings, although not numerous, were comprehensively reviewed within the last year [1,28]. The fact that melatonin promotes the deacetylation of mitochondrial SIRT3 and FOXO3a, which results in the stimulation of the antioxidant enzyme, SOD2, leaves unexplained the importance of the direct free radical scavenging component of melatonin in protecting this organelle and the cell as a whole from free radical damage; this issue requires resolution.

SIRT3 has multiple functions in mitochondria. It impacts the respiratory chain complexes and elevates ATP production, influences enzymes of the citric acid cycle, holds sway over amino acid metabolism and controls fatty acid oxidation. Additionally, SIRT3 affects mitochondrial dynamics [233,234,235,236,237]. Melatonin shares many of these same actions [231,238,239,240,241,242,243]. Whether melatonin’s effects on mitophagy, mitochondrial bioenergetics, fusion or fission are a consequence of its epigenetic actions may be a fruitful area of investigation.

Considering the multiple overlapping functions and their very long-standing cohabitation in mitochondria, it seems likely that SIRT3 and melatonin have interactions beyond regulating SOD2 activity. It is our prediction that they cooperate in the regulation of a variety of mitochondrial functions. We also expect these interactions will be clarified in the near future.

## Figures and Tables

**Table 1:** Experimental evidence that melatonin reduces oxidative stress and related events in cells, animals, and humans. Listed here are representative publications where melatonin was used to diminish oxidative damage and/or associated physiological/molecular changes following exposure to a variety of different chemical toxins/processes that normally lead to oxidative mutilation of critical molecules, cellular apoptosis, and compromised organ function. As mentioned, these are representative reports; there are many more. If the reader has a specific interest in a toxin/melatonin interaction, it (they) could be easily found in an appropriate literature search. Also, for many of the subjects listed, there are numerous publications documenting the protective function of melatonin, e.g., in ischemia/reperfusion injury, heavy metal toxicity, etc. The reports are in chronological order.

Toxin/Process	Tissue Protected	Reference
Carbon tetrachloride	Liver	[69]
Hyperbaric oxygen	Lung and brain	[70]
2-Nitropropane	Lipid and DNA	[71]
Cerulein	Pancreas	[72]
Quinolinic acid	Brain	[73]
δ-Aminolevulinic acid	Lipid and DNA	[74]
Neonatal asphyxia (human)	Lipid	[75]
Ochratoxin A	Liver and kidney	[76]
Ultraviolet radiation	Leucocytes	[77]
Respiratory distress syndrome (human)	Lung	[78]
1-Methyl-4-phenyl-1,2,3,6-tetrahydro pyridine	Brain	[79]
Organ transplantation	Liver	[80]
Diquat	Lipid	[81]
Cerebral hypoperfusion	Brain	[82]
Aspirin (human)	Gastrointestinal tract	[83]
*Opisthorchis viverrini* (liver fluke)	Liver	[84]
Methamphetamine	Brain	[85]
Doxorubicin	Heart	[86]
Cobra toxin	Multiple tissues	[87]
Strenuous exercise	Skeletal muscle	[88]
Ionizing radiation	Oral mucosa	[89]
Ischemia/reperfusion	Heart	[90]
Cadmium	Brain	[91]
Phosphine	Heart	[92]
Neonatal sepsis (human)	Neonate	[93]
ST-segment elevation, myocardial infarction (human)	Heart	[94]
Ischemia/reperfusion	Brain	[95]
*p*-Cresol	Mesenchymal stem cells	[96]
Arsenic trioxide	Liver	[97]
Type 2 diabetes	Cardiovascular system	[98]
Hexavalent chromium	Spermatogonia	[99]
Ischemia/reperfusion	Lung	[100]
